# FEM Analysis as a Tool to Study the Behavior of Methacrylate Adhesive in a Full-Scale Steel-Steel Shear Joint

**DOI:** 10.3390/ma15010330

**Published:** 2022-01-03

**Authors:** Marta Kałuża, Jacek Hulimka, Arkadiusz Bula

**Affiliations:** 1 Department of Structural Engineering, Faculty of Civil Engineering, Silesian University of Technology, Akademicka 5 St., 44-100 Gliwice, Poland; jacek.hulimka@polsl.pl; 2 Jacobs Engineering Group Inc., M. Konopnickiej 31 St., 30-302 Krakow, Poland; a.bula@o2.pl

**Keywords:** adhesive joint, methacrylate, numerical analysis, full-scale elements, DLJ

## Abstract

The use of adhesive to joint structural elements, despite many advantages of this technology, is not a method commonly used in engineering practice, especially in construction. This is mainly due to the poor recognition of the behavior, both in terms of testing and analysis, of joints made on a scale similar to the actual elements of building structures. Therefore, this paper presents the results of model tests and then numerical analyses of adhesively bonded joints made of high-strength steel elements in a full-scale (double-lap joint). In order to properly model the adhesive connection, material tests of the methacrylate adhesive were performed in the field of tensile, shear (in two versions: single lap joint test and thick adherent shear test) and bond properties. Comparison of the results of the model and numerical tests showed very good agreement in terms of the measurable values, which makes it possible to consider the results obtained in the adhesive layer as reliable (not directly measurable in model tests). In particular, the distribution of stresses inside the adhesive layer, the range of plastic zones and areas of loss of adhesion are presented and discussed. The results indicate the possibility of a reliable representation of the behavior of adhesively bonded joints of high-strength steel, thus providing a tool for the analysis of semirigid adhesive in large-size joints.

## 1. Introduction

The first structural adhesively bonded joints were employed as early as the first half of the twentieth century [1]. Following the research by Pierre Castan in 1936, which yielded epoxy resins as more than suitable for binding metals, Ciba started large-scale production of the resins in 1946. At first, epoxy resins were used mostly in the aeronautics industry [2,3]. Then they found their way into the automotive [4,5] and even construction industries [6]. The dynamic advancements in welded joints in the second half of the twentieth century led to the abandonment of adhesively bonded joints for steel structures. They were not researched or used at a significant scale. Still, they were applied for two types of joints. The first one was strengthening of structures using composite materials that are glued to the original structure [7,8,9]. The other group was multimaterial structures where mechanical point fastening was advised against, including glass structures (hybrid beams, prefabricated composites structures, façade elements) [10,11,12]. The most popular of these applications are epoxy adhesives, which are strong, stiff, and well researched in terms of necessary mechanical characteristics [13,14,15].

The authors’ original research [16,17,18] demonstrated that semi-rigid methacrylate adhesives offer much higher resistance of large-area lap joints on high-quality steel. The application of flexible adhesives facilitates stress redistribution over a larger joint distance than in joints with stiff epoxy adhesives. In contrast, the elastic-plastic characteristics of methacrylate adhesives result in a ductile failure. 

It is difficult or even impossible to conduct an in-depth analysis of the behavior of an adhesive layer on an actual joint tested in a laboratory. Therefore, a reliable numerical model of a sample may prove very useful. The literature offers numerous approaches to adhesively bonded joint modelling today. Still, each has to be adapted to the type of joint, adherend materials, and adhesive characteristics. 

There are three primary models proposed in FEA analyses to describe the adhesive layer [19,20]. The first one is the continuous model, which considers the adhesive’s stiffness based on Young’s modulus, Poisson’s ratio, and the thickness of the adhesive bond. The initiation of adhesive failure can be defined in two ways. For rigid materials, it is necessary to determine the conditions necessary for the occurrence of a crack and its propagation, which is in line with LEFM (Linear Elastic Fracture Mechanic). In such a case, the base is cohesive elements (in Cohesive Zone Modelling—CZM), which prevent any mesh refinement over the thickness of the adhesive layer. This approach works for thin adhesive bonds where no significant changes in stress distribution are found over the bond thickness. For flexible adhesives, the failure is defined with the criterion of plasticization of the adhesive layer. It is then modelled identically to the adherends (steel elements), which facilitates the refinement of the mesh over the thickness of the adhesive layer, and then analysis of any increases in and distribution of stress over the thickness. Regrettably, this adhesive layer modelling does not facilitate any failure mode analysis because it is only possible to determine the finite element within which the material degraded.

The continuous model based on LEFM is popular in modelling joints with relatively stiff adhesives. Campilho et al. illustrated the influence of CZM shape [21] and overlap length [22] on strength prediction of thin adhesive layer in a single-lap joint (SLJ) made of aluminum for different geometry and adhesive combinations (two epoxy adhesives: brittle and ductile, and ductile polyurethane adhesive). Gustfson and Waas [23] showed the influence of adhesive parameters on the outcome of cohesive zone finite element simulation. They conducted numerical analyzes for various joint configurations that are used to characterize joint parameters (SLJ, double-cantilever beam DCB and end notch flexure ENF). Katsivalis et al. [24] described the possibility of applying cohesive zone models for the prediction of damage and failure of DCB glass/steel joints glued using two different adhesives (epoxy and methacrylate).

The next method for modelling an adhesively bonded joint is to use interface relationships between the adherend and the adhesive layer. In the simplest terms, this approach can be employed for joints where the adhesive layer is so thin it can be considered as a thickness of zero. In such a situation, the macroscopic parameters of the adhesive are irrelevant. Fracture mechanics are used in analyses instead. What is important in this scenario is that the joint failure is always due to the loss of adhesion (adhesive failure). This modelling method for joining a member to the surface of reinforced concrete beams was presented by Sena-Cruz et al. [25]. The adhesive bond was considered an interface surface with adhesion properties of the adhesive. It is the optimal solution because the bond can be considered a zero-thickness layer in the context of the entire reinforced concrete element. A similar approach was adopted by Feito et al. [26] when modeling CFRP composite strips. The adhesive layer was considered an interface surface between composite matrices here as well, which yields a delamination.

For a more in-depth analysis of adhesives, interface relationships can be used to model parameters of adhesion of the adhesive to the adherend. This approach necessitates a limitation in the way the adhesive layer is modelled. The adhesive layer failure cannot be defined in accordance with LEFM, which is employed to model interface surfaces but has to follow the introduction of a criterion for plasticization (ductile adhesives). 

The most versatile adhesive layer model is provided by XFEM (eXtended Finite Element Method). It facilitates the modelling of adhesive bond failure by breaking links between nodes in the adhesive layer. It was achieved thanks to additional functions that describe node displacement during crack emergence until two new surfaces are created. This method describes such discontinuities as cracks, allowing them to propagate in a controlled manner. Additionally, when elements of XFEM are used, the type of the finite element does not need to be the same as in the continuous model, so it is possible to refine the finite element mesh over the bond thickness. This modelling method facilitates a more precise bond joint failure mode assessment, removing any restrictions on crack shape. Nevertheless, this tool is very time consuming and laborious as it requires a complex adhesive model and a huge amount of input data. Such an innovative approach on the XFEM to modeling the adhesively bonded joints is presented, among others, by Ramesh et al. [27] considering different criteria for damage initiation, and Santos and Campilho [28] using XEFM as a method to predict the joint’s behavior. These analyzes reflect the behavior of the same joints made of aluminum plates using three types of adhesive, as described in detail in [21,22]. 

The literature today offers many studies comparing basic models for building numerical adhesively bonded joints. An interesting summary of CZM and XFEM analysis results, indicating the capabilities and limitations when modelling SLJ and DLJ specimens is presented by Campilho et al. [29], and DCB specimens by Antunes et al. [30]. Struparu et al. [31] compared the behavior of the single-lap joint modeled using CZM and XEFM, and these results were related to the stresses measured during the optical measurement of the actual research model. 

Another noteworthy work is comparative analyses made by Kim et al. [32] and Sadegahi et al. [33]. Both research groups presented four different FE models based on fracture toughness criteria to modeling fractures on adhesively bonded single-lap joints. In the first paper a joint with three overlap lengths (10, 20 and 30 mm) made of unidirectional carbon fiber reinforced adherend with brittle epoxy adhesive was described; in the second one, the steel adherent joint was modeled using ductile epoxy adhesive with a thickness of 0.2 and 0.9 mm. An interesting proposal of a hybrid inverted method to analyze the adhesive interface stress at a composite single-lap bonded joints was presented in [34]. The data obtained from the optical measurement of the samples (displacement field distribution) were used here to create a reliable numerical model of the joint. 

All numerical analyses in the referenced literature focused on modelling simple, small-scale SLJ, DLJ, DCB, ENF joints or others [35]. These types of joints are used to determine basic material parameters of the adhesive alone, not structural joints tested on full-scale elements. In the case of large models, basic material parameters need to be analyzed in-depth first so that material models used can be as accurate as possible (full adhesive characteristics), and then the right models for the entire joint can be selected.

This paper focuses on the possibility of identification of adhesive behavior within a shear joint between full-scale steel elements. The point is that the adhesive cannot be observed directly because the adherends obscure the relatively thin layer. Therefore, any external observations (such as optical deformation measurements) are restricted to steel elements, and any conclusions regarding adhesive behavior are indirect. On the other hand, introducing any gauges, such as strain gauges, into the adhesive layer, disturbs its continuity, resulting in observer error whereby the test alone affects its result. In this case, the problem can be resolved with the right models for numerical simulation of the investigated phenomenon. 

The model joint is made of high-strength steel flat bars joined with a methacrylate adhesive, which has great potential for steel bonding. The first part of the paper presents detailed material tests of the steel and adhesive with a discussion of the results in reference to parameters in the product data sheet (taking into account differences in standards). Next, the authors present a method for modelling the adhesively bonded joint, which reflects the actual given full-scale DLJ. Thanks to the right adhesive model based on its actual measured parameters, the structural model is reliable, which was confirmed in a comparison of the model and numerical test results (to the available extent). Hence, it was concluded that the consistency with parameters determined with the test demonstrated the reliability of adhesive behavior in the FEM analysis. The last part of the paper presents an analysis of the adhesive layer behavior, including its plasticization and consecutive stages of adhesive failure. 

## 2. Laboratory Material Testing—Results and Discussion

### 2.1. Steel

The steel specimens were made of steel Domex 700 and were tested according to PN-EN ISO 6892-1:2016 recommendation [36]. Three identical specimens were tested at a speed of testing of 1 mm/min. The elongation of the specimens was measured using extensometers. The primary mechanical properties of the steel are given in Table 1.

### 2.2. Adhesive

All material tests were performed with the Plexus MA 420 adhesive (Shannon, County Clare, Ireland) [37]. This adhesive is a two-component methacrylate recognized as a semi-rigid material for structural joints. Based on the detailed material tests described below, the main tensile, shear and adhesion parameters were determined.

#### 2.2.1. Tensile Strength

**Figure 1:**
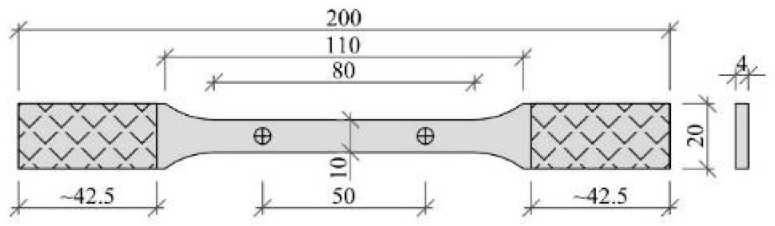
Geometry of tested specimen.

In order to determine the basic properties of the methacrylate adhesive, standard tensile tests were carried out. Quasi-static tensile tests were performed on typical dog-bone shape samples (Figure 1) according to PN-EN ISO 527 recommendations dedicated for plastics [38,39]. Three series of five repetitions were performed at speeds of testing of 1, 10 and 100 mm/min. The samples were fixed in custom made aluminum clamps with a base grip length of 42.5 mm. 

**Figure 2:**
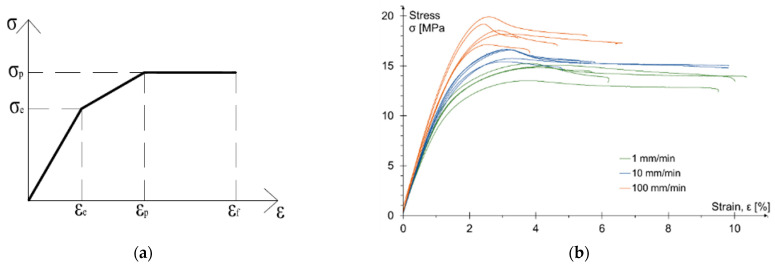
Tensile test: (**a**) simplified model for elastic-plastic material; (**b**) the stress-strain relationship obtained for three test speeds.

The elongation of the samples was measured using extensometers, and in some samples using an optical measurement system (DIC). In order to determine reliable parameters for the elastic-plastic material model (Figure 2a) important characteristic points—elastic stage of work, yielding and final failure—were specified as averages for each of the considered test speeds. The laboratory tests results are presented in Table 2. Additionally, in the strain range of 0.05–0.25%, according to [38], the modulus of elasticity was calculated. The stress-strain relationships for all three test speeds are given in Figure 2b. The mean value of the Poisson’s ratio for the standard test speed (1 mm/min) amounted to 0.365. Some detailed information is presented in [40,41].

The results indicate a significant influence of the load speed on the adhesive strength. Faster load increase results in greater strength and stiffness of the adhesive, with a simultaneous significant reduction in plastic deformation.

#### 2.2.2. Shear Strength—Single Lap Joint (SLJ)

**Figure 3:**
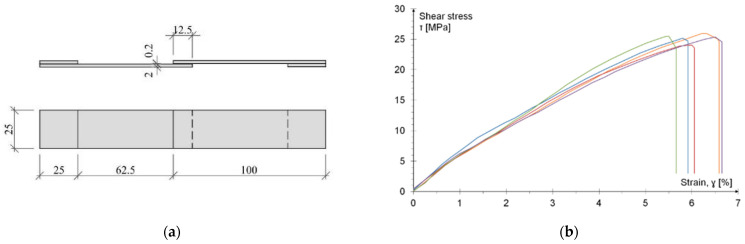
Single-lap joint test: (**a**) geometry of the tested specimen; (**b**) stress-strain relationship (indirectly determined). The line colors were assigned to the next five identical samples.

The determination of the shear strength of the adhesive was performed on samples subjected to tension in the single lap joint (SLJ) scheme, in accordance with PN-EN 1465:2009 [42] recommendation. Aluminum flat bars of 1050A alloy with a modulus of elasticity of 69 GPa and a yield point of 120 MPa (information according to the manufacturer’s data) were used. The plates were prepared directly before bonding by sandblasting, cleaning and coating with the primer recommended by the adhesive manufacturer. The joint geometry is shown in Figure 3a. In the test, aluminum plates were adopted as adherent because the adhesive parameters provided by the manufacturer [37] were also tested using aluminum elements.

During the test, aluminum flat bars became plasticized in the area of the glued joint. Thus, an incorrect mode of failure was obtained because no adhesive or cohesive failure in the adhesive layer was observed. Of course, in the next loading steps the failure of the adhesive occurred, but it was a secondary phenomenon. Therefore, it can be concluded that since the metal elements were not broken, the glue was responsible for the final destruction (however, with the wrong mode of failure—“metal failure”). 

Measurement of flat bar elongation (with the use of extensometers) allowed for indirect determination of the shear stresses in the adhesive. The values of displacements measured directly in the jaws of the machine were reduced by the values taken from the extensometers. In this way, the stress-strain characteristic were obtained (Figure 3b). There was no yielding of the adhesive layer. The shear strength was calculated as the product of the tensile force and the area of the adhesive joint, and the average value amounted to 25.22 MPa (COV 2.5%) with an average deformation of 6% (COV 5.7%). 

Of course, one has to take into account the fact that the value of the shear strength depends on the type of adherents.

#### 2.2.3. Shear Strength—Thick Adherent Shear Test (TAST)

**Figure 4:**
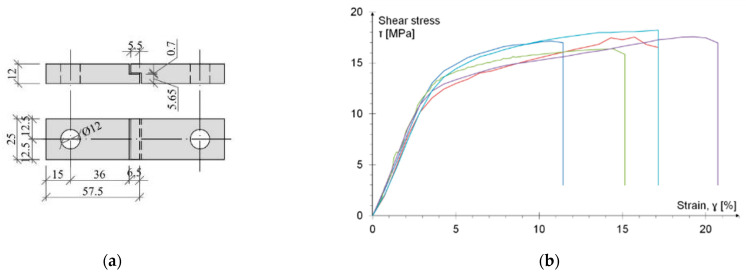
TAST test: (**a**) the geometry of tested specimen; (**b**) the stress-strain relationship (indirectly determined). The line colors were assigned to the next five identical samples.

The typical single-lap shear test of analyzed adhesive did not allow determination of its fill characteristic due to the occurrence of an undesirable mode of specimen failure. Therefore, it was decided to perform a pure shear adhesive test according to the ISO 11003-2:2001 recommendation [43] in so-called TAST (Thick Adherent Shear Test). The specific geometry of the tested sample (Figure 4a), “thick adherent” and a special gripping system allows measurement of shear stresses without significant impact of additional peeling stresses (no bending effect appears). Specimens were mounted by steel blocks with diameter 10 mm, passing through pin holes. Five repetitions were performed at a speed of testing of 0.5 mm/min. Figure 4b presents the stress-strain relationships.

The result, shown in Figure 4b, allows separation of two phases of the adhesive work: elastic and plastic. The shear strength was calculated as the product of the tensile force and the area of the adhesive joint. The elastic limit is noted for an average shear stress of 13.49 MPa and 4.5% average strain (with COV amounted to 4.5% and 6.1%, respectively). After reaching this value the plastic phase (with strengthening) begins. The failure of the adhesive occurred at the average stress of 17.36 MPa (with COV amounted to 3.4%). In the non-elastic phase, at the failure significant strains ranging from 10.7% to 19.2% were observed. A large range of the failure strains is probably due to the automatic shutdown of the hydraulic press with a certain sequence of instantaneous results. 

In each case, the mode of failure indicated an adhesive-cohesive character.

The secant shear modulus was also calculated, the mean value of which amounted to 379 GPa (with COV amounted to 4.1%). 

#### 2.2.4. Bond Strength

**Figure 5:**
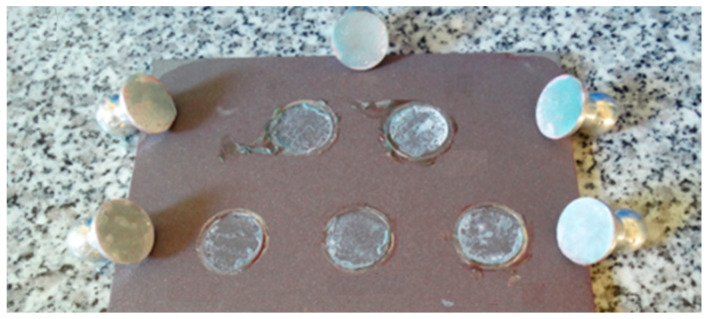
The mode of failure of tested specimens.

In order to determine the bond properties of the MA420, a pull-off test of the adhesive to steel surface was carried out. This test was made according to the PN-EN ISO 4624:2016 [44] recommendation. The test consisted of measuring the adhesion force between an aluminum stamp with a diameter of 20 mm and a properly prepared surface. The test was performed on steel plates (S235JR) with dimensions of 150 × 150 × 6 mm, which were sandblasted, degreased with acetone and painted with a dedicated primer immediately before bonding the measuring stamps. During the test, measurements were made on seven aluminum measuring stamps. The thickness of the adhesive layer was 0.1 mm. As a result of the test, an average adhesion of 21.85 MPa (COV 2.0%) was obtained, and the failure was of an adhesion nature (Figure 5). 

#### 2.2.5. Analysis and Discussion of Material Test Results

The tensile and shear properties of the methacrylate adhesive—Plexus MA 420—provided by the manufacturer in the technical sheet [37] are summarized in Table 3. The technical sheet of the product defines the standards on the basis of which tensile properties—ASTM D638 [45] and shear properties—ASTM 1002 [46] were determined. 

A comparison of the obtained results from own laboratory tests (point 2.2.1) with the data from the technical sheet (Table 3) shows large discrepancies in values, especially in the deformation parameters (Young’s modulus and elongation at rupture).

The standard [45] specifies as many as five types of sample geometry depending on the type of the tested plastic (rigid, semirigid or nonrigid) and the thickness of the sample. Additionally, it defines different load speeds for the selected sample type, while requiring to “select the lowest speed that produces rupture in 0.5 to 5 min. for the specimens’ geometry”. If the manufacturer of the adhesive does not provide detailed information on the type of sample and at what speed it was tested, it is not possible to reliably compare the obtained strength and deformability values of the adhesive. 

**Figure 6:**
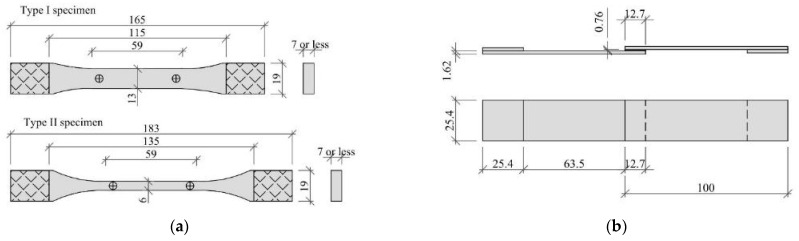
Geometry of the tested specimen: (**a**) according to standard [45]; (**b**) according to standard [46].

However, methacrylic adhesive is considered to be semi-rigid plastic, so the shape of the test sample corresponds to Type I (material thickness 7 mm or less) or Type II (material does not break in the narrow section with the preferred Type I specimen). The geometry of these samples is shown in Figure 6a, and is very similar to the sample tested according to the standard [38]. However, the thickness of the sample tested according to the standard it is not known [45]. 

The analysis of the test speed of Type I and II samples, in terms of possible sample elongation (assuming that the failure will occur between 0.5 and 5 min of the test), indicates that the tested samples were loaded with a speed of 50 mm/min. This gives a real elongation between 25 mm and 250 mm. Therefore, assuming the distance between grips of 115 and 135 mm for Type I or II sample, respectively, this results in elongation ranging from 30 to 50% within this range. At a speed of 5 mm/min, the maximum elongation would be just over 25 mm (which is only 22% of the length of the Type I sample and 19% of the length of the Type II sample) and, at a speed of 500 mm/min, the analyzed sample would have to extend from about 250 to 2500 mm, which is unrealistic with respect to its dimensions.

When analyzing the values given in the product sheet, it is puzzling why there are large ranges of values for most of the parameters specified. For tensile strength it is over 10%, with an E-modulus of over 30%, with strain at failure of over 65% and with shear strength about 26% (in relation to lower values). Since the relevant standard defines at what speed dog bone-shaped pieces should be tested, the values given are not the result of different loading speeds. Similarly, the values of temperature and humidity, as well as the conditions of sample seasoning, are strictly established. Thus, under strict conditions, the cited significant dispersions seem to indicate high instability of the material, which was not observed in own study, in which the results were consistent. Therefore, if one wants to clearly assume the characteristics of the adhesive for numerical analyses or theoretical considerations, it is necessary to rely on the results of one’s own tests performed on samples taken from the same batch of adhesive which was used to make the models. What is also puzzling in the analysis of the data sheet of the adhesive is the differences between the data contained in the sheets from 2018 and 2006, despite referencing the same standard [45]. 

As already mentioned, after analyzing the possible geometry of the sample and the loading speed, it must be concluded that the adhesive tensile strength test results quoted in the data sheet were obtained at a loading speed of 50 mm/min. Thus, the lowest value described there deviates slightly upwards from the values obtained in our tests. This may be due to the slightly different geometry of the samples, including the unknown thickness, which is only known to be no greater than 7 mm. Meanwhile, the thickness of the samples in our own tests was 4 mm.

With regard to the deformability of the adhesive, i.e., the modulus of elasticity and elongation at failure, the tested adhesive is much more rigid than declared in the product’s technical sheets. The differences obtained are probably due to the slightly different geometry of the samples, including their thickness. However, in view of the good repeatability of the results of our own tests the deformation data entered in the product sheets seem to be too optimistic. 

With regard to shear strength, the geometry of the joint specified in standard [46], and shown in Figure 6b, is almost identical to that specified in standard [42]. The difference is the thickness of the adhesive layer. In the tests carried out according to [42], it was equal to 0.2 mm and in the case of tests carried out according to [46], it was equal to 6 mm, while the ASTM standard for aluminum bonded components allows different joint thicknesses ranging from 0.015” (0.38 mm) to 0.120” (3.05 mm). 

In our own tests (Section 2.2.2), it turned out that the adhesive thickness required by standard [42] was insufficient to obtain the correct model failure, namely a failure in the adhesive layer (adhesive or cohesive failure). Therefore, it was considered that the result obtained was not reliable, although it was within the range of the values obtained by the manufacturer. What appears puzzling in this case, too, is the considerable dispersion of the values declared by the manufacturer of the adhesive. 

A reliable shear strength value for the tested adhesive was obtained using the TAST method [43]. In this method, the thickness of the adhesive layer was 0.7 mm and the joint length was 5.5 mm. This test allowed us to determine the maximum strength in the elastic phase as 13.49 MPa and at the time of failure (after plasticization), 17.36 MPa. This value is less than that given in the technical data sheet, but this difference is due to the quite different geometry of the joint and the different test method.

## 3. Laboratory Model Testing—Results and Discussion

### 3.1. Testing Procedure

**Figure 7:**
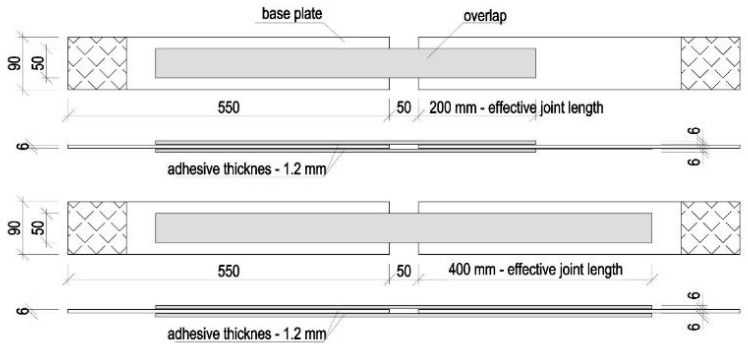
Geometry of the full-size adhesively bonded joints (DLJ).

The evaluation of the effectiveness of the structural shear joint of steel elements with methacrylate adhesive was performed on a double-lap joint, the geometry of which was adapted to the actual geometry of the structural elements. The DLJ specimens corresponded to the dimensions of the projected geometry of the overlays strengthening the steel load-bearing elements of load handling equipment and their supporting structures. Two types of DLJ specimens were made that differed in the effective length of the adhesive joint amounted to 200 and 400 mm (specimens with 200/400 mm and 400/400 mm overlap length). The specimens consisted of basic steel plates with the dimensions of 90 × 6 × 550 mm and both-sided overlaps glued to the basic plates. The cross-section of the overlaps was 50 × 6 mm and their length was 850 mm and 650 mm, depending on the type of specimen. The connection between the steel components was provided by a layer of methacrylate adhesive with a thickness of 1.2 mm. Three specimens of each type were made from one portion of the adhesive. A view of the tested specimens is given in Figure 7.

**Figure 8:**
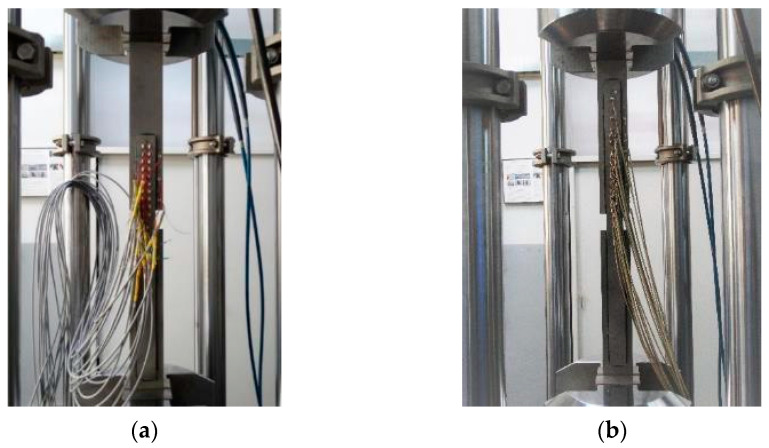
Specimens prepared for tests with a traditional measurement system (strain gauges): (**a**) model with an effective length of 200 mm; (**b**) model with an effective anchorage length of 400 mm.

The specimens were tested under axial tension in one cycle up to failure. The loading speed was 1.27 mm/min (0.05 inch/min), which was adopted from the ASTM D3528-96 recommendation [48]. Figure 8 shows the elements tested. 

During the test, the tension force, elongation of the specimen and strains on the steel surface (at selected points of the overlap) were measured.

### 3.2. Results and Discussion

The detail test results are included in [16]. Only the most important results of the basic model tests performed are discussed here.

It should be explained that in the full laboratory tests four specimens of each type were made, while the fourth model in a given series was made later with the use of glue from another portion. These additional specimens were used to perform optical measurement of deformation using the Digital Image Correlation method (DIC). Due to the fact that slightly different characteristics were obtained (which was explained by slightly different parameters of the glue, as in [16]), the results of additional models were omitted in this paper because the material tests were performed for the portion of glue used to make the basic specimens (three of each type). 

**Figure 9:**
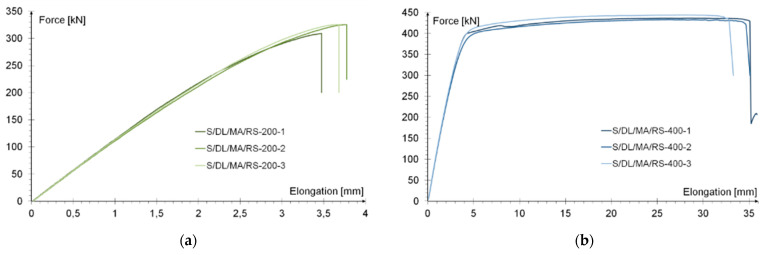
Load-elongation relationship in (**a**) specimens with an effective anchorage length of 200 mm; (**b**) specimens with an effective anchorage length of 400 mm.

The behavior of the tested specimens is illustrated by the relationship between the tensile force and elongation of the tested elements, as shown in the Figure 9a,b.

**Figure 10:**
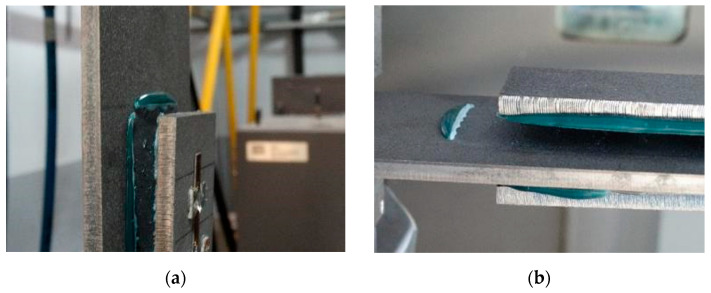
Specimens after failure: (**a**) model with an effective anchorage length of 200 mm; (**b**) model with an effective anchorage length of 400 mm.

A significant influence of the effective joint length on the behavior of the specimen was observed. The use of a joint with a length of 200 mm resulted in the element as a whole working in an almost elastic manner until the joint was damaged. This occurred at an average force of 319 kN and a strain (averaged over the length of the specimen) of 0.46%. In all tested specimens, “adhesive failure” was noted, so the connection between the adhesive and the surface of the steel base element was damaged (Figure 10a).

In elements with an effective joint length of 400 mm, two work phases were distinguished: elastic and plastic. The yielding of the specimens took place at an average force of 403 kN, and the strain averaged over the length of the sample was 0.52%. During the plastic phase, the elongation of the samples increased very rapidly. At failure, it exceeded 30 mm, which corresponded to the average strain of the models amounting to 3.73%. The force during yielding process increased slightly and at the time of failure amounted to ca. 437 kN. Measurement of the length of steel elements after failure allowed for the conclusion that the steel base elements had plasticized, which was also confirmed by subsequent tests in which the optical method of measuring deformation of steel surfaces was used [16]. Unfortunately, due to the limitations of the measurement methods, it cannot be clearly determined to what extent the adhesive had plasticized. The damage of the joint was carried out in two stages. From the point of view of the ASTM standard [48], at the moment of steel yielding the joint was damaged because one of the system components was theoretically destroyed (sudden increase in deformation). So, “metal failure” occurred in this convention. However, after the steel yielded, the joint deformation continued to increase, most likely because the adhesive in this area began to work in the plastic range, which included successive sections of the adhesive joint. This situation lasted until the bond between the adhesive and steel surface was broken, which can be considered a secondary adhesive failure (Figure 10b). Figure 10b shows the model after final damage with a very large elongation of the base steel plate (difference between the edge of the overlay and the adhesive residues on the base plate) resulting from the plasticization of the steel and probably the adhesive joint. The above description was based on indirect premises and, describing the phenomenon in a qualitative sense, it was not possible to quantify it.

## 4. Numerical Modeling—Assumption

### 4.1. Model Selection

Analysis of the behavior of the test models, as well as the mode of their failure, enables a reasonable adoption of the most appropriate numerical model of the adhesive joint. Point 1 presents theoretical descriptions of the adhesive models that are most commonly used in numerical modeling. 

Considering the assumed thickness of the adhesive layer (1.2 mm) and the guidelines of the standard [38], the methacrylate adhesive analyzed can be classified as a semi-rigid material [49] because its thickness is between the values accepted for rigid (0.1–0.2 mm) and fully deformable (3–4 mm) adhesives. 

The force-elongation relationship shown in Figure 9b indicates plasticization of the adhesive layer and the failure pattern of the models (primary or secondary) in each case studied indicates adhesive failure of the joint between the steel surface and the adhesive layer (Figure 10a,b). 

Taking into account the above considerations, in the numerical analyses performed the methacrylate adhesive layer is modeled as a continuum with contact layers. The plasticization criterion for the adhesive was adopted based on the results of research [50], in which it was shown that in the case of acrylic adhesives the best convergence of results was obtained for the Drucker-Prager linear plasticization criterion. Due to the fact that methacrylate adhesives, such as acrylic adhesives, have a high deformability before failure, a decision was made to use this criterion as well. Contact layers (zero thickness) between the adhesive bond and the steel components were modeled by applying adhesive characteristics to the bonded surfaces and by applying a contact model using the LEFM. This approach allowed for crack propagation in contact layers. For this purpose, the criterion of maximum nominal stresses was adopted and, after reaching the limit stress in a specific place, the contact was lost and the crack propagated. A simplification in the calculation model was that the crack could only increase in the contact layer; there was no such possibility in the material itself (adhesive or steel). The steel components were described by a material model with elastic-plastic characteristics, according to the Huber-Misses criterion.

ABAQUS [51] software was used for modeling the DLJ specimens.

### 4.2. Material Model of the Adhesive

#### 4.2.1. True Values

The basis for determining the material parameters of the adhesive were the tensile results for dog-bone-shaped samples (point 2.2.1) and TAST results (point 2.2.3). An identical approach can be found in other studies [52]. The values obtained in material tests (engineering approach) do not take into account the influence of transverse deformations of samples appearing during the laboratory test. The adopted Drucker-Prager model, on the other hand, is based on the actual (true) values of stresses and strains; therefore, the obtained results were transformed [38]. 

True tensile stress and strains are calculated according to the guidelines of the National Physical Laboratory [53]. The following equations express elastic stress, elastic strain and transverse elastic strain respectively:

(1) $$\sigma_{T}=\frac{\sigma^{\prime }{}_{T}}{{\left(1-v^{\prime }{\varepsilon^{\prime }}_{T}\right)}^{2}}\;,$$

(2) $$\varepsilon_{T}=\ln\left(1+{\varepsilon^{\prime }}_{T}\right),$$

(3) $$\varepsilon_{t}=\ln\left(1+{\varepsilon^{\prime }}_{t}\right).$$

The values marked with ′ are values obtained directly from the tensile test (Section 2.2.1), i.e., engineering values. 

From the tensile test, the E-modulus and Poisson ratio were measured to calculate the following plastic strain, transverse plastic strain and plastic Poisson’s ratio (the values with ‘ are taken from the laboratory tests):

(4) $$\varepsilon_{T}^{P}=\varepsilon_{T}-\ln\left(1+\frac{\sigma_{T}}{E}\right),$$

(5) $$\varepsilon_{t}^{P}=\varepsilon_{t}-\ln\left(1-v^{\prime }\frac{\sigma_{T}}{E}\right),$$

(6) $$v^{P}=-\frac{\varepsilon_{t}^{P}}{\varepsilon_{T}^{P}}.$$

True shear parameters are calculated on the basis on TAST results (point 2.2.3). The plastic strain, effective shear stress and effective shear plastic strain are expressed by the equations given below:

(7) $$\varepsilon_{S}^{P}=\varepsilon_{S}-\frac{\sigma_{S}}{G},$$

(8) $$\sigma_{s,eff}=\sqrt{3}\sigma_{S},$$

(9) $$\varepsilon_{s,eff}^{P}=\frac{\varepsilon_{S}^{P}}{\sqrt{3}}.$$

**Figure 11:**
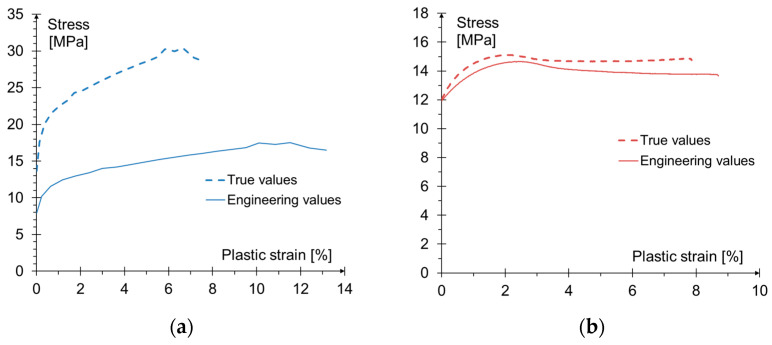
Comparison of engineering (from laboratory tests) and true values determined for: (**a**) shear stresses; (**b**) tensile stresses.

Figure 11a,b show the comparison of stress-strain relationships in the plastic range for the results obtained directly from the laboratory tests (engineering values) and the values needed in the numerical model (true values).

#### 4.2.2. Continuous Model with Plasticization

In the Drucker-Prager material model, the plasticization criterion (*f_DP_*) is expressed as the relationship:

(10) $$f_{DP}=t_{DP}-d_{DP}-p_{DP}\cdot \tan\left(\beta_{DP}\right)=0$$

where *t_DP_* is the effective stress defined as a component of the principal stress, *d_DP_* is a cohesion, i.e., a material parameter related to plasticizing stresses at shearing, *p_DP_* is the mean hydrostatic stress, *β_DP_* is the material internal friction angle, and tan(*β_DP_*) is the plasticity coefficient for hydrostatic stress. 

The plasticity coefficient is determined on the basis of two stress states, tensile and shear, and is determined from Equation (11):

(11) $$\tan\beta_{DP}=\frac{3\cdot \left(\lambda_{DP}-1\right)}{\left(\lambda_{DP}+1\right)}$$

where *λ_DP_* is a hydrostatic stress sensitivity parameter defined as:

(12) $$\lambda_{DP}=\frac{\sqrt{3}\cdot \left(\sigma_{S}/\sigma_{T}\right)}{2-\sqrt{3}\cdot \left(\sigma_{S}/\sigma_{T}\right)}$$

True tensile stress, $\sigma_{T}$, and shear stress, $\sigma_{S}$, used in Equation (12), must correspond to the same value of plastic strain:

(13) $$\sigma_{T}\cdot \varepsilon_{T}^{P}=\sigma_{S}\cdot \varepsilon_{S}^{P},$$

As long as the stresses do not reach the limit determined by Equation (10), the adhesive model is based on linear elasticity, using the relationship between the Young’s modulus and the elastic Poisson’s ratio of the material. After exceeding this limit, plastic deformation begins to increase. The increase depends on the flow parameter *ψ_DP_*. This coefficient should be determined on the basis of the plastic component of the Poisson’s ratio *v_p_*, from the equation:

(14) $$\tan\left(\psi \right)=\frac{3\cdot \left(1-2\cdot v_{p}\right)}{2\cdot \left(1+v_{p}\right)},$$

**Figure 12:**
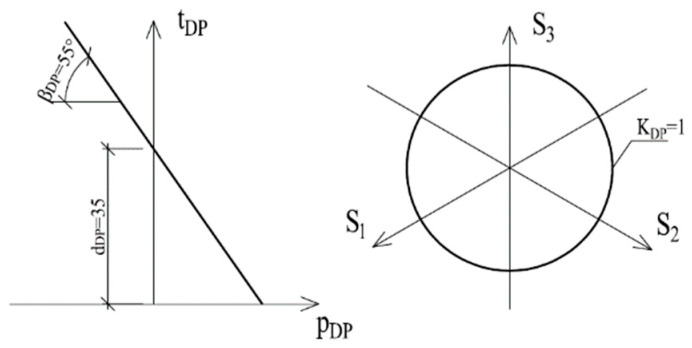
Material model with parameters for the adhesive.

Based on the transformed values (true values given in Figure 11) and Equations (11) and (14), the parameters necessary to characterize the material model of the adhesive were determined. The values used in the numerical analysis for Drucker-Prager linear plasticization criterion are shown in Figure 12. 

### 4.3. Material Model of the Steel

**Figure 13:**
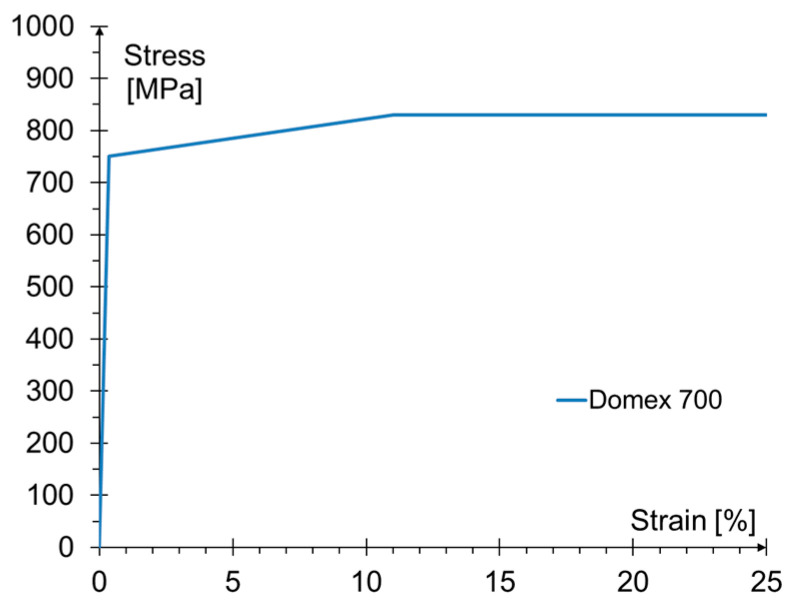
Material model of the steel.

The material characteristics of the steel adopted as the elastic-plastic model are presented in Figure 13 based on the parameters listed in Table 1 (point 2.1). No steel failure criterion was adopted, but it was assumed that after exceeding the limit stresses, the deformation would increase rapidly, which would allow determination of plasticization in the steel element. 

### 4.4. Contact Layer Model

A contact layer, with zero thickness between the adhesive and the steel elements was modeled by conferring bond characteristics to the surfaces to be joined. Based on the test results (point 2.2.4), the limit bond stress was assumed to be 21.85 MPa, which is also the limit shear stress. This represents destruction in the adhesive form without significantly damaging the adhesive layer itself. Therefore, it was decided to use the pull-off strength to determine the adhesion parameters of the adhesive contact layers. 

As a criterion for the initiation of degradation, the criterion of maximum nominal stresses was adopted according to Equation (15), where successive *t_i_*^0^ denote the limit values of tensile and shear stresses. This approach is similar to that described in [54].

(15) $$\mathrm{MAX}\left(\frac{\langle t_{n}\rangle }{t_{n}^{0}};\frac{t_{s}}{t_{s}^{0}};\frac{t_{t}}{t_{t}^{0}}\right)=1.$$

**Figure 14:**
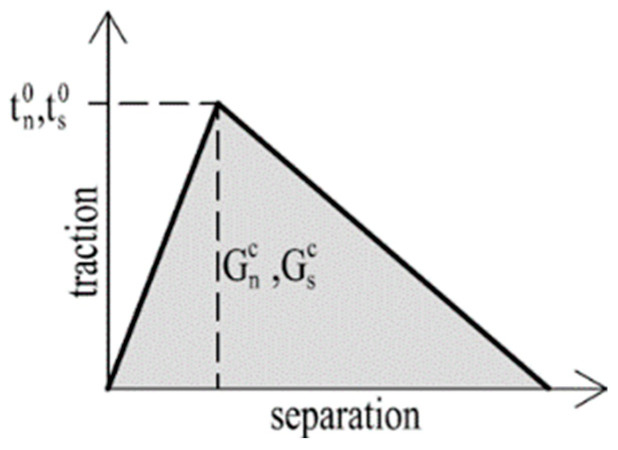
Triangular shape for fracture energy.

The fracture energy was assumed to be 3.5 kJ/m^2^ for tensile (G_n_^c^) and shear (G_s_^c^) based on the study [54]. This describes the evolution of crack propagation and, according to the study [55], a triangular shape was selected (Figure 14). Due to the deformable adhesive layer, cracks occur in the peel force region of connection so this assumption is a good approximation. When the fracture energy is used/dissipated, a fracture occurs in a given region.

## 5. Numerical Model—Results and Discussion

### 5.1. Characteristic of the Numerical Model

**Figure 15:**
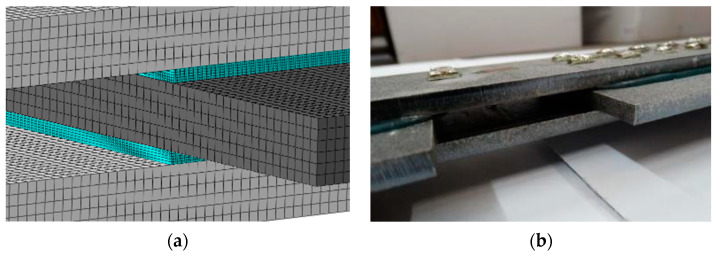
View of the joint in the middle of the specimen: (**a**) numerical model; (**b**) laboratory test.

**Figure 16:**
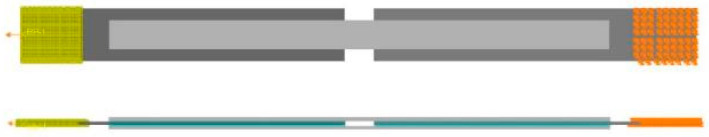
The static model of DLJ adopted in numerical analysis.

The numerical model of the joint was made in a three-dimensional state of stress with the use of finite elements of the C3D8 type (eight-node cubic elements). The dimensions of the finite element were assumed to be 1.0 × 1.0 × 1.0 mm in both types of steel plates and 0.25 × 0.25 × 0.25 mm in the adhesive layer (Figure 15). The static scheme and the mode of loading of the joint were adopted identically to the model laboratory tests. A stiff connection at one end of the specimen and a stiff link with one independent reference point (along which the load occurs) of the second end were assumed. Specimens with two different effective joint lengths (200 and 400 mm) were modeled [40]. Figure 16 shows the adopted static scheme of a DLJ specimen.

### 5.2. Nmerical Model Validarion—Discussion

**Figure 17:**
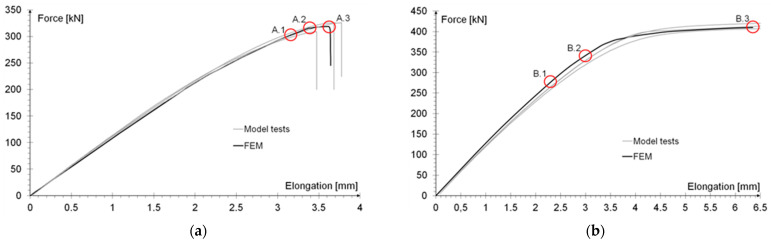
Comparison of force-elongation relationships from laboratory tests and numerical analysis for specimens with effective joint length of: (**a**) 200 mm; (**b**) 400 mm.

Analysis of the validity of the modelled double-lap joint was carried out by comparing the force-elongation relationship for both types of joints analyzed [40]. Figure 17 shows the graphs obtained from laboratory tests of three models (made using the same batch of adhesive) and a numerical model based on the results of tests of adhesive from the same batch. Each time, three displacement values were assumed, for which the force values measured in the tests and read from the numerical model were compared. These points were designated as A.1, A.2, and A.3 for the sample with an effective anchoring length of 200 mm and B.1, B.2, and B.3 for the sample with an effective anchoring length of 400 mm.

For both types of specimens, a very good agreement of the analyzed results was obtained (Table 4), which allows us to conclude that the performed numerical model reflects the actual behavior of the tested samples very well. This consistency was obtained both in the elastic phase of the models’ work and during the plasticization of the specimens with an effective joint length of 400 mm. 

The slightly overestimated resistance value obtained in the models with the anchoring length of 400 mm is probably due to the inaccuracy of the adhesive bond in the model joints, where the excess adhesive was squeezed out of the pads and formed a local thickening of the joint (Figure 10b). As a result, the actual active area during shearing was slightly larger than the theoretical one, and the edge stress distribution was smoother than in the idealized numerical model.

In the numerical model with an anchoring length of 400 mm, as in the laboratory tests, a clear increase in displacement was obtained when a tensile force of 400 kN was reached. This was due to the sum of the effect of elongation of the base flat bar at the jaws of the testing machine (stress in the steel exceeded the proportional limit) and plastic deformation in the adhesive layer. The visible difference between the numerical model and the tested samples in this phase was due to the adopted bilinear material model of steel. In reality, however, steel plasticization occurs in a gentler manner than in the simplified model, so that a higher stiffness of the numerical model immediately before steel plasticization is observed in the graphs. In addition, the rheology of the adhesive itself in the plastic state may also have influenced the results.

### 5.3. Deformation Analysis—Discussion

In standard laboratory tests, only a certain amount of measurement data can be obtained, usually with a certain number of points. In this specific given case, these measurements were limited to the places where deformations were measured (strain gauge alignment) or, in the case of optical measurements, to the accessible surfaces of the steel components. Any results concerning the adhesive bond can only be estimated indirectly. Hence, having a reliable numerical model of the joint allows fast and efficient execution of deformation analysis and determination of the full stress distribution in the components, including the adhesive bond layer. It is also possible to simultaneously analyze the adhesion of the adhesive to the surface of the steel components and the behavior of the steel itself.

**Figure 18:**
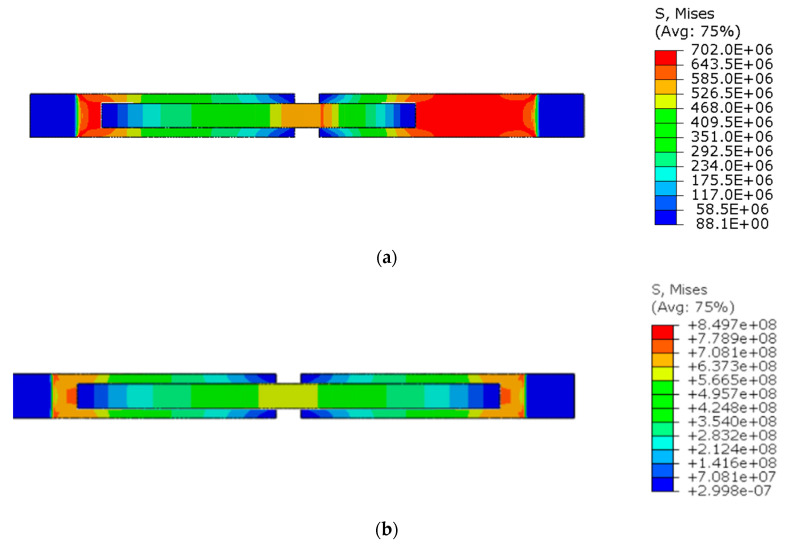
Tensile stresses in numerical models with overlap lengths of: (**a**) 200/400 mm; (**b**) 400/400 mm.

Figure 18 shows the stress distribution obtained from the numerical analysis on the surface of the overlap with effective joint lengths of 200 mm and 400 mm. The maps show the stress values at the time of theoretical failure of the numerical models (point A.3 with tensile force of 318.6 kN; point B.3 with tensile force of 406.2 kN). The colors in the individual graphs are scaled independently and do not correspond to each other.

For the sample with the overlap length of 200/400 mm, when the joint resistance (ultimate tensile force) was reached, the stresses in the steel components were within proportional limits, so that the diagram of the relationship between the force and the elongation of the steel components should be linear. However, in Figure 9a, in the last phase of work of the component it can be seen that there has been some minor plastic deformation in the model as a whole, suggesting plasticization of the adhesive prior to the failure. However, the above conclusion is indirect and cannot be quantified on the basis of the data obtained in the model tests. At the same time, a visual inspection of the destroyed samples (Figure 10a) indicates that the final failure of the tested models was adhesive in nature at the contact surface between the adhesive and the 90 × 6 mm base flat bar. During the preliminary analysis of the obtained laboratory results [17], it was found that an anchorage length of 200 mm was assumed to be insufficient to provide efficient bonding between high-strength steel components. The specimen failure occurred earlier than the joined components load-bearing capacity expired. Only the numerical analysis that was carried out provided complete knowledge of the joint behavior and enabled the conclusion that the anchoring length was ideal, as it did not allow plasticization and hence excessive elongation of the sample (which is disadvantageous from the point of view of practical application). 

Table 5 shows the adhesive bond behavior determined in the numerical analysis at the analyzed points in the model with an overlap length of 200/400 mm. In the adhesion maps, a value of 0.00 indicates full adhesion and 1.00 indicates no adhesion. On the other hand, for the plasticity of the adhesive, a range from 0.00 to 1.00 indicates the relative level of plasticity of the adhesive (1.00 is full plasticity).

An analysis of the plasticity level of the adhesive shows that initially (A.1) the plasticization of the adhesive bond was almost complete. Due to the possibility of further stress buildup in the plastic working range of the adhesive, there was a gradual loss of adhesion between the base flat bar (90 × 6 mm) and the adhesive layer (red zone), initially in the area of the joint edge in the center of the sample. At the same time, the joint retained full adhesion to the overlap (50 × 6 mm). As the zones where the adhesion of the main flat bar was lost increased, there was a decrease in the extent of the plastic zone of the adhesive, as the adhesive ceased to transmit stress in the areas of adhesive failure. Although adhesive failure (between adhesive and the surface of the base plate) is ultimately visible, in reality the mechanism is complex and can be described as adhesive failure with partial plasticization of the adhesive. In the case of the overlap, only a local loss of adhesion occurring in the last phase, i.e., just before the joint failure, was observed. Confirmation of the described behavior of the adhesive layer is provided by the photograph of the damaged sample (Figure 10a), where the loss of adhesion between the adhesive and the surface of the base flat bar is clearly visible. The plasticization of the adhesive described herein explains the aforementioned slight nonlinearity of the model elongation-force diagram.

**Figure 19:**
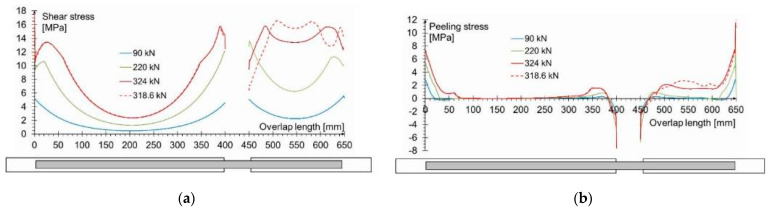
Stresses in the adhesive layer taken from numerical model: (**a**) shear stresses (**b**) tensile (peeling) stresses.

In order to better illustrate the stress distribution in the adhesive layer (obtained in numerical analysis) for the specimen with 200/400 mm length of overlap, the shear and tensile peeling-stress distributions in the adhesive layer (Figure 19a,b respectively) are additionally shown in the axis of the tension specimen. Only selected force levels are shown in the diagrams for greater clarity. From a qualitative point of view, it is significant that moments before the failure (ca. 312 kN) the shear stresses reached practically the same value (15.5 MPa) on the short sections of both joints in the central part of the model. Over the length of the shorter joint, this showed a decrease in stress at the ends and a shift in the maximum value slightly towards the center of the joint. A further small increase in load in the direct failure phase (up to 318.6 kN) did not cause any visible changes in the stress distribution in the longer joint, while in the shorter joint a decrease in stress at one of its ends was clearly visible, accompanied by a shift of the highest stress values (up to 15.5 MPa) towards the center of the joint length. The two red lines along the length of the shorter joint also showed equal shear stress values between their extreme peaks, which indicates plasticization of the adhesive. This behavior is desirable as it allows the adhesive to work properly over the entire length of the joint (which, of course, is due to the inherent flexibility of the adhesive layer, which is a function of the modulus of elasticity and the thickness of its layer). The values of the shear stresses obtained at the failure, in the range from 15.5 MPa to 16.5 MPa, are similar to those obtained in our TAST tests; the difference is due to the joint size in the material test and in the joint model.

As can easily be seen in Figure 19a, the shear stress distribution along the length of the two contact surfaces (adhesive layers) is very similar, with pronounced peaks near their ends and a drop in the value as the center of the length of each contact surface is approached. Clearly, the stresses in the shorter joint (200 mm) are higher than in the longer joint (400 mm) for the same force value.

In the case of the distribution of tensile (peeling) stresses as shown in Figure 19b, they were clearly concentrated at the ends of both joints, whereas in the failure phase, they reached values of up to 7 MPa (i.e., significantly below the tensile strength from the material tests). Only in the failure phase did tensile stresses appear along the entire length of the shorter joint (but in a negligible value of up to about 2 MPa), while they were practically equal to zero along most of the longer overlap.

The distribution of shear stresses presented in Figure 19a is identical to the stress map shown in Figure 18a. Of course, the stress map is made for one selected force value, hence it is general in nature, while Figure 19 shows successive changes in the stress distribution during loading (in selected loading steps) showing the graphs for several selected load values.

It should be noted that the obtained shear stress distribution along the joint length was almost identical to that for the lap-type joints, despite a significant difference in the size of the adhesively bonded joint. However, with much larger surfaces of actual glue joints the differences in the stress values along the length of the joint are greater and strongly depend on the deformability of the glue and its layer thickness. In general, the stiffer the adhesive and the thinner its layer, the more incorrect the assumption of uniform stress distribution in the adhesive layer. Thus, in an actual lap joint, the load-bearing capacity is determined by the maximum local shear stresses, and not the average value along the joint length (as in SLJ test and TAST).

An analogous analysis was performed for an effective joint length of 400 mm. In this case, maps of the plastic work of the adhesive and of the plasticization of the base plate (90 × 6 mm) were most important. The results are shown in Table 6. At a force of about 270 kN (point B.1), which is well before the visible plasticization of the entire model, local plasticization of the adhesive layer occurred at both ends of the contact surface between the overlap and the base flat bar. A further increase in force, up to point B.2 (ca. 333 kN), led to the development of a zone of plasticization of the adhesive, which at that time covered almost the entire surface of the joint. Such a condition is responsible for the onset of a clearly nonlinear character of the diagram showing the relationship between the force and the elongation of the sample visible in Figure 9b. At a force of about 406 kN (which corresponds to a stress in the basic flat bar of just over 750 MPa), there was a loss of proportionality between the steel deformation and the stress, and consequently a huge increase in specimen elongation at a virtually constant force. 

Thus, the deformation of the steel in the base flat bar starts to be responsible for the nonlinear nature of the diagram (as a result of its narrowing, the stresses continue to increase, and the yield point is exceeded). This failure of the models is also adhesive in nature, but the direct cause is plasticization of the steel in the base flat bar, and the loss of adhesion of the adhesive to the steel itself is secondary. It can be assumed that the use of steel with even higher performance characteristics would result in a higher load-bearing capacity of the joint, for which only the adhesive bond would be responsible.

## 6. Conclusions

This study considered the possibility of obtaining detailed data on the behavior of a sheared adhesive joint connecting steel components in a double-lap joint. Since direct observations of the behavior of such a joint do not allow for measuring the accessible surfaces of the steel components, and since the introduction of strain gauges directly into the adhesive layer disrupts its work, in the model tests inferences concerning the behavior of the adhesive in the joint can only be made indirectly. Meanwhile, by using appropriately advanced numerical modeling based on reliable characteristics of the adhesive confirmed by tests (and, of course, the characteristics of the materials being joined), it is possible to obtain a direct image of the behavior of the adhesive in the joint, as well as to assess the contact phenomenon at the interface between the adhesive layer and the joined materials. 

Based on the performed model tests and the numerical analysis, the following conclusions can be formulated. 

(1) The basis for creating a reliable numerical model is to perform detailed tests on the mechanical characteristics of the adhesive. This is because the data contained in the technical data sheets for adhesives are not always reliable, even if they are based on the same standards as our own tests. In addition, the final results alone (regarding strength, elongation, and other characteristics) are not sufficient to adopt a simplified model of the adhesive because it is usually necessary to know all characteristics of the behavior of the material under load. 

(2) In the case of methacrylate adhesive (and probably other adhesives), the loading speed has a significant effect on the strength and elongation test results; this is important given the wide range of test speeds allowed by the standards. 

(3) In model tests of adhesive joints, data obtained from strain gauge measurements at selected points in the models are insufficient to fully describe their behavior. 

(4) In the case of the methacrylate glue tested, reliable results were obtained using the continuous model with plasticization criterion for the adhesive, and the contact layers were assumed to have zero thickness, with the bond characteristics given. It should be noted that the above criteria are specific to adhesives with significant deformation prior to failure (semi-rigid material) and therefore cannot be considered universal. 

(5) The results of numerical analysis allowed for detailed mapping of the behavior of the adhesive layer under the load of the tested models, including the analysis of the extent of the adhesive joint plasticization and the areas of loss of adhesion. Thus, the possibility of a reliable assessment of the adhesive joint behavior under load, and a description of the course of the final failure of the models, was demonstrated. At the same time, plastic failure of steel in 400 mm long models, previously inferred only indirectly in tests with local strain gauge measurements, was demonstrated. 

Additionally, general conclusions were formulated based both on the described laboratory tests and on our own and other research.

(6) Attention should be paid to the influence of the thickness of the adhesive layer on the test results. In the case of a joint with a small area (SLJ, TAST), an increase in shear strength is visible in the case of a thinner adhesive layer, which results from the cooperation of the entire, short joint (it is even included in the standards, where the failure force is divided by the entire surface of the joint, thus assuming identical stresses at each point). In turn, in large joints, it is desirable to have an adhesive layer thick enough to ensure proper interaction of the adhesive (plasticization) along the length of the joint. Hence, there is a need to individually select the thickness of the adhesive layer in a given joint, depending both on the dimensions of the joint and the stiffness of the adhesive itself.

(7) The mode of failure of the adhesively bonded joint (adhesive, cohesive or mixed) depends on the ratio between the adhesion at the contact of the adhesive with adherents and the cohesion inside the adhesive layer. In our laboratory tests, each time the adhesion was damaged this could be explained by the use of an adhesive with relatively medium deformability. In the case of brittle adhesives, one can expect cohesive failure, but the final method of failure will always depend on the mutual correlation of a number of factors, such as the adhesive elasticity modulus, the thickness of its layer, the joint surface and method of surface preparation of adherents, and also on the test conditions (e.g., temperature).

As our main conclusion, it should be stated that a properly performed numerical analysis of an adhesively bonded joint, based on appropriate material tests, is a reliable tool to study the behavior of an adhesive throughout the model loading process. 

Of course, one has to be aware that the described tests are only fragmentary and are limited to two types of models made of the same steel and joined with the same adhesive. Thus, the conclusions of the tests are not universal, especially in terms of material models, which must be adapted each time to the characteristics of the adhesive and the bonded materials.

## Figures and Tables

**Table 1:** The mechanical parameters of steel.

	Proportional Limit Stress [MPa]	Yield Strength [MPa]	Tensile Strength [MPa]	Ultimate Elongation [%]
Average values	~750	791.7	825.8	21.5
COV [%]	–	0.6	1.1	5.4

**Table 2:** Tensile properties of the adhesive.

Test Speed [mm/min]	Elastic Strength σ_e_ [MPa]	COV [%]	Elastic Strain ε_e_ [%]	COV [%]	Yield Strength σ_p_ [MPa]	COV [%]	Yield Stain ε_p_ [%]	COV [%]	Failure Strain ε_f_ [%]	COV [%]	E-modulus [MPa]	COV [%]
1	8.0	7.5	0.8	25.0	14.7	4.1	4.1	7.3	8.4	23.8	1058	5.9
10	11.0	5.4	1.2	33.3	16.2	3.1	3.2	3.1	6.8	27.9	1131	2.1
100	13.9	4.3	1.4	21.4	18.3	3.8	2.4	4.1	4.6	39.1	1224	4.7

**Table 3:** Tensile and shear properties of adhesive.

Data	Test Speed [mm/Min]	Tensile Strength [MPa]	E-Modulus [MPa]	Elongation at Rapture [%]	Shear Strength [MPa]
Technical data	50 ^1^	18.6–20.6	517–689	30–50 ^2^	20.7−26.2 ^2^ (SLJ)

^1^ According to ASTM D638 [45], tests can be made at speeds of 5, 50 and 500 mm/min (samples type I and II)—explanation in the text below. ^2^ Values taken from the datasheet from 2018 [37], i.e., during the material testing period. In the datasheet from 2006 [47] the elongation to at rupture amounted to 100–125% and shear strength was 12.0–15.5%.

**Table 4:** Tensile and shear properties of adhesive.

	Model with 200/400 mm Overlap			Model with 400/400 mm Overlap		
	A.1	A.2	A.3	B.1	B.2	B.3
	3.10 mm	3.35 mm	3.63 mm	2.31 mm	2.95 mm	6.00 mm
Laboratory tests						
Mean value [kN]	299.0	311.2	319.7	264.1	323.1	411.2
COV [%]	1.1	1.4	2.3	1.1	1.5	1.3
Numerical model						
Value [kN]	298.5	314.0	318.6	270.2	332.6	406.2
Difference	0.17%	0.90%	0.34%	2.31%	2.94%	1.22%

**Table 5:** Tensile and shear properties of adhesive.

	Points Given in Figure 14a		
	A.1	A.2	A.3
Loss of adhesion: adhesive—base plate (90 × 6 mm)	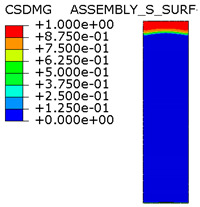	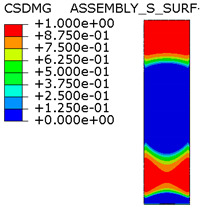	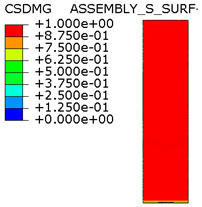
The plastic phase of the adhesive layer and base plate	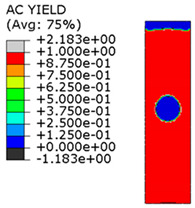	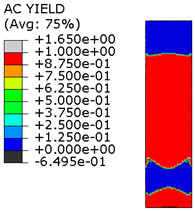	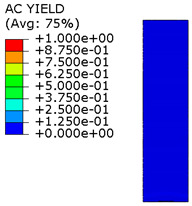
Loss of adhesion: adhesive—overlap (50 × 6 mm)	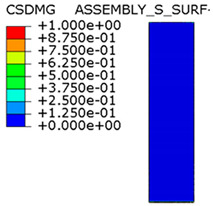	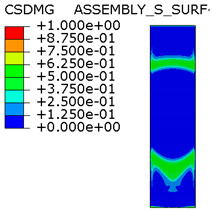	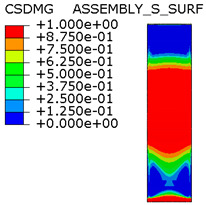

**Table 6:** Tensile and shear properties of adhesive.

	Points Given in Figure 14b		
	B.1	B.2	B.3
The plastic phase of the adhesive layer and base plate (90 × 6 mm)	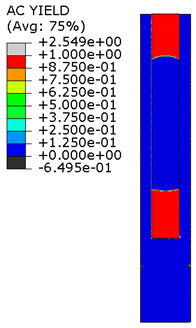	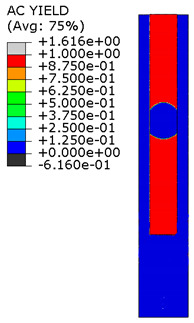	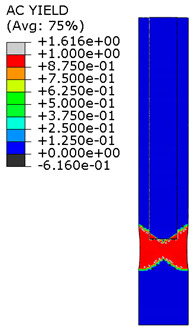

## Data Availability

Detailed data is available in [40].
